# Deregulation of lncRNA HIST1H2AG-6 and AIM1-3 in peripheral blood mononuclear cells is associated with newly diagnosed type 2 diabetes

**DOI:** 10.1186/s12920-021-00994-z

**Published:** 2021-06-06

**Authors:** Hui Jiang, Peian Lou, Xiaoluo Chen, Chenguang Wu, Shihe Shao

**Affiliations:** 1 Department of Endocrinology, Affiliated People’s Hospital of Jiangsu University, Zhenjiang, 212002 China; 2 Xuzhou Center for Disease Control Prevention, Xuzhou, 221000 China; 3 School of Medicine, Jiangsu University, 301 Xuefu Road, Zhenjiang, 212013 Jiangsu Province China

**Keywords:** Long noncoding RNA, Type 2 diabetes mellitus, Gene expression, Microarray analysis, Lnc-HIST1H2AG-6, Lnc-AIM1-3

## Abstract

**Background:**

Type 2 diabetes mellitus (T2DM) is mainly affected by genetic and environmental factors; however, the correlation of long noncoding RNAs (lncRNAs) with T2DM remains largely unknown.

**Methods:**

Microarray analysis was performed to identify the differentially expressed lncRNAs and messenger RNAs (mRNAs) in patients with T2DM and healthy controls, and the expression of two candidate lncRNAs (lnc-HIST1H2AG-6 and lnc-AIM1-3) were further validated using quantitative real-time polymerase chain reaction (qRT-PCR). Spearman’s rank correlation coefficient was used to measure the degree of association between the two candidate lncRNAs and differentially expressed mRNAs. Furthermore, the KEGG (Kyoto Encyclopedia of Genes and Genomes) pathway and GO (Gene Ontology) enrichment analysis were used to reveal the biological functions of the two candidate lncRNAs. Additionally, multivariate logistic regression analysis and receiver operating characteristic (ROC) curve analysis were performed.

**Results:**

The microarray analysis revealed that there were 55 lncRNAs and 36 mRNAs differentially expressed in patients with T2DM compared with healthy controls. Notably, lnc-HIST1H2AG-6 was significantly upregulated and lnc-AIM1-3 was significantly downregulated in patients with T2DM, which was validated in a large-scale qRT-PCR examination (90 controls and 100 patients with T2DM). Spearman’s rank correlation coefficient revealed that both lncRNAs were correlated with 36 differentially expressed mRNAs. Furthermore, functional enrichment (KEGG and GO) analysis demonstrated that the two lncRNA-related mRNAs might be involved in multiple biological functions, including cell programmed death, negative regulation of insulin receptor signal, and starch and sucrose metabolism. Multivariate logistic regression analysis revealed that lnc-HIST1H2AG-6 and lnc-AIM1-3 were significantly correlated with T2DM (OR = 5.791 and 0.071, respectively, both *P* = 0.000). Furthermore, the ROC curve showed that the expression of lnc-HIST1H2AG-6 and lnc-AIM1-3 might be used to differentiate patients with T2DM from healthy controls (area under the ROC curve = 0.664 and 0.769, respectively).

**Conclusion:**

The profiles of lncRNA and mRNA were significantly changed in patients with T2DM. The expression levels of lnc-HIST1H2AG-6 and lnc-AIM1-3 genes were significantly correlated with some features of T2DM, which may be used to distinguish patients with T2DM from healthy controls and may serve as potential novel biomarkers for diagnosis in the future.

**Supplementary Information:**

The online version contains supplementary material available at 10.1186/s12920-021-00994-z.

## Background

Diabetes mellitus (DM), one of the most common chronic diseases worldwide, is characterized by elevated blood glucose levels. According to a report by the International Diabetes Federation (IDF), the prevalence of DM in the global population aged 20–79 years is expected to rise from 8.8% in 2015 to 10.4% in 2040. By 2040, the number of patients with DM in the world will increase by 54.7% [1]. China has the world’s largest diabetes epidemic; one study estimated that among adults in China, the overall prevalence of diabetes was 10.9% and that of prediabetes was 35.7% in 2013 [2]. Type 2 DM (T2DM) reportedly accounts for > 90% of all patients with DM [3]. T2DM has become an important public health problem that has serious effects on quality of life due to chronic complications, including nephropathies, retinopathies, neuropathies, and cardiovascular diseases [4]. Therefore, exploring its occurrence and developmental mechanism is important for early diagnosis and treatment of T2DM. In previous decades, noncoding RNAs (ncRNAs), involving microRNAs, small interfering RNAs (siRNAs), long noncoding RNAs (lncRNAs), circular RNAs (circRNAs), and piwi-interacting RNAs (piRNAs) have been the focus of research because of their important biological functions that are strongly associated with the occurrence and development of many diseases, including T2DM [5, 6].

LncRNAs are defined as a kind of functional RNA molecule (larger than 200 nucleotides) without the ability of protein coding, but with the functions of chromosome modification, transcriptional regulation, and post-transcriptional processing [7]. LncRNAs appear to be the main regulators of gene expression and govern diverse biological processes of genomic imprinting, metabolism, proliferation, cell differentiation, immune regulation, and apoptosis [8, 9]. Several studies have focused on the relationship between lncRNAs and various diseases, including diabetes, cardiovascular diseases, neurological diseases, and cancer [10–14]. The abnormal expression of lncRNAs in T2DM has recently been shown in the analysis of the human *β*-cell transcriptome; lncRNAs can regulate the secretion of insulin by the islet *ß* cells and might play a role in the regulation of glucose metabolism by the liver and skeletal muscles. Maternally expressed gene 3 (MEG3), taurine upregulated gene 1 (TUG1), and the growth inhibition of specific transcription (GAS5) regulate islet *ß* cells in the synthesis and secretion of insulin through PDX1 and MafA [15–17]. Zhang et al. found that lncRNA H19 in the liver of mice on a high-fat diet was increased and liver-specific overexpression of H19 promoted the production of liver glucose, causing hyperglycemia and insulin resistance (IR), whereas the systemic knockout of H19 increased the inhibitory effect of insulin on hepatic gluconeogenesis [18]. Brown fat lncRNA1 (Blnc1) could promote the differentiation of brown fat. Furthermore, the inhibited expression of Blnc1 in adipose tissue aggravates obesity-related brown fat whiteness and adipose tissue inflammation, leading to more severe IR [19].

Several studies have shown that lncRNAs were closely related to diabetes; however, the abnormal expression and function of lncRNAs in T2DM remain largely unknown [15–19]. Thus, the purpose of this study was to investigate the association of novel lncRNAs with T2DM and to determine potential biomarkers by comprehensive analysis, including microarray analysis (lncRNA and mRNA expression profiles), large-scale validation, multivariate logistic regression analysis, and ROC curve analysis.

## Methods

### Ethics and subjects

The study was conducted within the Chinese Han population in Jiangsu Province, China, and approved by the Institutional Ethics Committee of the Affiliated People’s Hospital of the Jiangsu University, Zhenjiang, Jiangsu, China (K-20170109-Y). Informed consent was provided by all the participants. One-hundred newly diagnosed patients with T2DM (45 men and 55 women; mean age: 49.2 years, range: 18–70 years) were enrolled from the endocrinology clinic at the Affiliated People’s Hospital of the Jiangsu University (Zhenjiang, Jiangsu, China) from December 2017 to May 2018. For the pupose of this study, the diagnosis of T2DM was established according to the World Health Organization (WHO) diabetes diagnostic criteria published in 1999 [20], which defined diabetes based on a fasting plasma glucose concentration of 7.0 mmol/L (126 mg/dL) or higher, a 2-h postload plasma glucose concentration of 11.1 mmol/L (200 mg/dL) or higher, or a random plasma glucose concentration of 11.1 mmol/L (200 mg/dL) or higher. Ninety healthy controls (43 men and 47 women; mean age: 46.5 years, range: 18–70 years) were recruited from the medical examination center. None of the participants had been treated with any hypoglycemic agent. Those with a history of type 1 diabetes, other special-type diabetes, cancer, pregnancy, immunodeficiency, chronic organ disease, infectious disease, or chronic or acute complications of diabetes were excluded.

### Anthropometric and biochemical measurements

For all participants, blood samples were collected from the cubital vein after an overnight fast of at least 10 h. Body mass index (BMI) was calculated as weight (kg) divided by the square of the height (m). Blood pressure was measured in a calm state.

Total cholesterol (TC), triglyceride, high-density lipoprotein cholesterol (HDL-C), low-density lipoprotein cholesterol (LDL-C), blood glucose, and uric acid (UA) levels were measured using AU5800 Clinical Chemistry System (Beckman Coulter AU5800, Brea, CA, USA). The white blood cell (WBC) count was assessed using an automatic blood cell analyzer (Beckman DxH 500, Brea, CA, USA). The human insulin levels were measured using Access Immunoassay System (Beckman Coulter Unicel DxI 800, Brea, CA, USA). The glycosylated hemoglobin A1c (HbA1C) level was tested using HLC-723G8 (Tosoh Corporation, Tokyo, Japan). Homeostatic model assessment-IR (HOMA-IR) was determined according to the following formula: [fasting plasma glucose (mmol/L) × fasting insulin (lU/mL)]/22.5. Further, homeostatic model assessment of *β*-cell function (HOMA-B) was determined as follows: [20 × insulin (lU/mL)/(fasting plasma glucose (mmol/L) − 3.5]%.

### Microarray analysis

Total RNA was extracted from the peripheral blood mononuclear cells (PBMCs) of patients with and without diabetes using the MiniBEST Universal RNA Extraction Kit (TaKaRa, Tokyo, Japan), as per the manufacturer’s instructions. RNA integrity was assessed using Agilent Bioanalyzer 2100 (Agilent Technologies, Santa Clara, CA, USA), and the purity was measured using a NanoDrop2000 spectrophotometer (Thermo Scientific, MA, USA). The satisfactory RNA samples [OD260/280 > 1.8, OD260/230 > 1.5, and RNA Integrity Number (RIN) ≥ 8.0] were taken for further microarray analysis. Finally, RNA samples from two patients with T2DM and two healthy controls were selected for chip analysis (OEBiotech, Shanghai, China). Sample labeling (labeled with cy3), microarray hybridization, and washing were performed as per the manufacturer’s standard protocols. Typically, total RNA was transcribed to double-strand complementary DNA (cDNA) and then synthesized into complementary RNA (cRNA). After that, second-cycle cDNA was synthesized from cRNA, followed by fragmentation and biotin labeling. The second-cycle cDNA was hybridized onto the microarray. After washing and staining, the arrays were scanned using an Affymetrix Scanner 3000 (Affymetrix, Santa Clara, CA, USA).

### Identification of differentially expressed genes

Affymetrix Gene Chip Command Console Software (version 4.0, Affymetrix) was used to extract the raw data signals. Expression Console Software (version 1.3.1, Affymetrix) was used to offer log scale robust multiarray analysis normalization for both gene and exon level analysis. GeneSrping Software (version 13.1; Agilent Technologies) was used to perform normalization and subsequent data processing. Differentially expressed genes (DEGs) were identified as significantly changed with the threshold of a fold-change (FC) of ≥ 2.0 (up or down) and a *P*-value of ≤ 0.05.

### Quantitative real-time polymerase chain reaction analysis

PBMC samples from each subject (90 healthy controls and 100 patients with T2DM) were separated from the whole blood using a red cell lysis buffer. Trizol reagent (Invitrogen, Carlsbad, CA, USA) was used to separate the total RNA. After separation, 10 mM of dNTPs, 2 μg of total RNA from each sample, 10 μM of random hexamers, 80 U of RNase inhibitor, and 200 U of MMLV reverse transcriptase (MBI Fermentas, Hanover, USA) were used and reverse transcribed into single-stranded cDNA; the samples were then stored at − 20 °C.

Two candidate lncRNAs (lnc-HIST1H2AG-6, lnc-AIM1-3) were quantified using quantitative real-time polymerase chain reaction (qRT-PCR) because of their differential expression (*P* < 0.05) (Table 1). Primer sequences used in this study were synthesized by Shanghai Sunny Biotechnology Co., Ltd. (Shanghai, China) and appended as follows: lnc-HIST1H2AG-6: Forward 5′-AGAAGTGAGATGTAACCAGAG-3′, Reverse 5′-ACTGTCTTTGAGGAACTGAC-3′; lnc-AIM1-3: Forward 5′-TGCCATAAGATTCTAACCTCTGC-3′, Reverse 5′-GGGTAAGTGGAAATTCTTTGTCC-3′; GAPDH: Forward 5′-AGGTGAAGGTCGGAGTCAAC-3′, Reverse 5′-GGGTGGAATCATATTGGAACA-3′. The reverse transcription was performed using 2 × SYBR Green mix (TransGen Biotech, China), and the qRT-PCR was performed using the ABI 7500 Fast RT-PCR System (Applied Biosystems, California, USA), the cycle was set at 95 °C for 5 min, followed by 40 cycles at 95 °C for 30 s, 58 °C for 30 s, 72 °C for 32 s, and 75 °C for 32 s to collect fluorescence. The abundance of each lncRNA transcript was calculated by analyzing the expression of housekeeper gene glyceraldehyde-3-phosphate dehydrogenase (GAPDH) using 2^−ΔΔCT^ method [21].

**Table 1:** Differential expression of selected lncRNAs identified using microarray analysis in PBMC between T2DM and controls

GeneSymbol	P value	FC (abs)	Regulation	NonCodeID	Chr
lnc-HIST1H2AG-6	0.0057	4.754367	Up	NONHSAT108316	chr6
lnc-AIM1-3	0.0228	2.151183	Down	NONHSAT114227	chr6

### Biological function analysis of lnc-HIST1H2AG-6 and lnc-AIM1-3

To better understand the biological function of lnc-HIST1H2AG-6 and lnc-AIM1-3, Spearman’s rank correlation test was performed first to screen their degree of association with differentially expressed mRNAs and construct an interaction network by Cytoscape software (version 3.7.0). Furthermore, KEGG Pathway and GO enrichment analysis were performed using the R software clusterProfiler package to explore the potentially enriched pathway and biological processes of the two lncRNAs related to mRNAs.

### Statistical analyses

Categorical data (including sex) were presented as numbers and percentages. Other data were presented as mean ± standard deviation (SD) values or medians with interquartile ranges. The normality of distributions was assessed for the variables. Differences between groups were evaluated using the unpaired Student’s t-test for data with normal distribution and the Mann–Whitney U test for data with abnormal distribution; *χ*^2^ test was used for categorical variables. Multivariate logistic regression analysis was used to analyze the association of T2DM with different variables, including age, BMI, systolic blood pressure (SBP), LDL-C, HDL-C, lnc-HIST1H2AG-6, and lnc-AIM1-3. The diagnostic value of lncRNAs was evaluated using the receiver operating characteristic (ROC) curve and area under the ROC curve (AUC). SPSS version 20.0 software was used for statistical analysis. *P*-values were two-sided, and *P*-values of < 0.05 were considered significant. The correlations of lnc-HIST1H2AG-6 and lnc-AIM1-3 with glucose metabolism indexes were assessed using Pearson correlation test.

## Results

### Main characteristics of the study cohort

A total of 190 subjects, including 90 controls and 100 patients with T2DM, were recruited in the present study. The main clinical characteristics of patients with and without diabetes are presented in Table 2. There were no significant differences in the distribution of sex, age, diastolic blood pressure (DBP), WBC, triglyceride, and UA between patients with T2DM and healthy controls (*P* > 0.05). However, the BMI, SBP, fasting plasma glucose (FPG), HbA1c, fasting insulin (FIN), HOMA-IR, HOMA-B, LDL-C, and HDL-C of patients with T2DM were significantly different from those of healthy controls (*P* < 0.05 or *P* < 0.01), indicating that these blood samples from the subjects described above could be used for the following experimental analysis.

**Table 2:** General characteristic of T2DM and control group for validation study

Clinical characteristics	T2DM (n = 100)	control (n = 90)	P value
Sex (male/female)	45/55	43/47	0.701
Age (years)	49.2 ± 12.2	46.5 ± 10.4	0.302
BMI (kg/m^2^)	24.2 (22.9–25.9)	20.8 (20.1–24.5)	0.047
DBP (mmHg)	78 (70–80)	73 (68–80)	0.124
SBP (mmHg)	138 (130–140)	130 (125–140)	0.003
WBC (× 10^9^/L)	4.8 (4.4–5.7)	4.7 (4.5–5.8)	0.984
FPG (mmol/L)	10.7 (8.8–13.2)	5.3 (4.9–5.8)	0.000
HbA1c (%)	9.3 (8.0–11.5)	4.8 (4.4–5.1)	0.000
FINS (uIu/ml)	5.3 (3.7–8.3)	7.2 (6.7–8.7)	0.000
HOMA-B	11.7(6.5–24)	108.8(91.6–141.9)	< 0.001
HOMA-IR	2.5(1.6–4.1)	1.6(1.4–2.0)	< 0.001
TG (mmol/l)	1.7 (1.0–2.3)	1.2 (0.9–1.8)	0.278
LDL-C (mmol/l)	2.8 ± 0.8	2.5 ± 0.5	0.002
HDL-C (mmol/l)	1.1 ± 0.3	1.3 ± 0.3	0.000
UA (mmol/L)	336.1 ± 59.5	330.5 ± 79.5	0.576

Data are expressed as mean ± standard deviation values, numbers, or medians (interquartile ranges). Data were analyzed using the unpaired Student’s t-test, x^2^-test and Mann–Whitney test, as appropriate

BMI, body mass index; DBP, diastolic blood pressure; SBP, systolic blood pressure; WBC, white blood cell; FPG, fasting plasma glucose; HbA1c, haemoglobin A1c; FIN, fasting insulin; TG, triglyceride; LDL-C, low density lipoprotein cholesterol; HDL-C, high density lipoprotein cholesterol; UA, urine acid

### Expression profile of lncRNA and mRNA by microarray analysis

In the present study, we compared the gene expression profile displayed by PBMCs from patients with T2DM with that of healthy controls by using the GeneSpring Software. The microarray cohort was composed of two patients who were newly diagnosed with T2DM and two healthy controls. There were 55 lncRNAs differently expressed in patients with T2DM compared with that in healthy controls; the detailed information was listed in Additional file 1. Among these 55 lncRNAs, 18 were upregulated and 37 were downregulated. In addition, we also identified 36 mRNAs that had aberrant expression between patients with T2DM and healthy controls; among these, 16 mRNAs were upregulated, and 20 mRNAs were downregulated. The detailed information about these 36 differently expressed mRNAs is presented in Additional file 2.

### Validation of two candidate lncRNAs

**Fig. 1:**
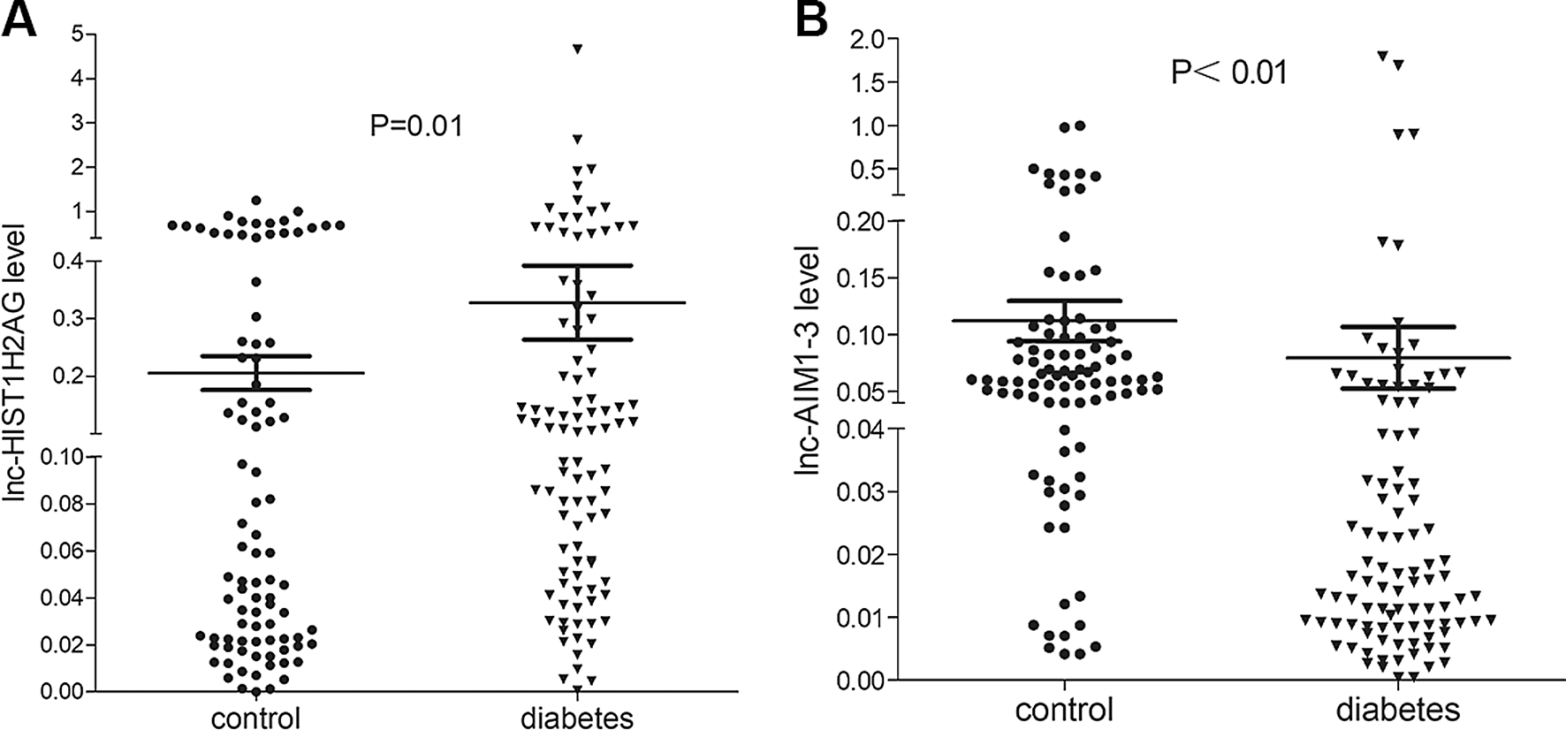
Relative expression levels of lnc-HIST1H2AG-6 (**A**) and lnc-AIM1-3 (**B**) in patients with T2DM and controls. The transcript levels were evaluated using qRT-PCR

Two candidate lncRNAs (lnc-HIST1H2AG-6 and lnc-AIM1-3) were chosen from the differentially expressed lncRNAs (FC ≥ 2.0 or ≤  − 2.0, *P* < 0.05) for further large-scale validation (90 controls and 100 patients with T2DM). QRT-PCR analysis of the blood samples of T2DM and healthy controls was performed. As shown in Fig. 1, compared with that in healthy controls, the HIST1H2AG-6 transcript level in patients with T2DM was significantly upregulated (*P* = 0.01), whereas the expression of AIM1-3 was significantly downregulated (*P* < 0.01), which were consistent with the results of microarray analysis, indicating the reliability of our data and the potential role of two candidate lncRNAs in T2DM.

### Biological function analysis of the two candidate lncRNAs

**Fig. 2:**
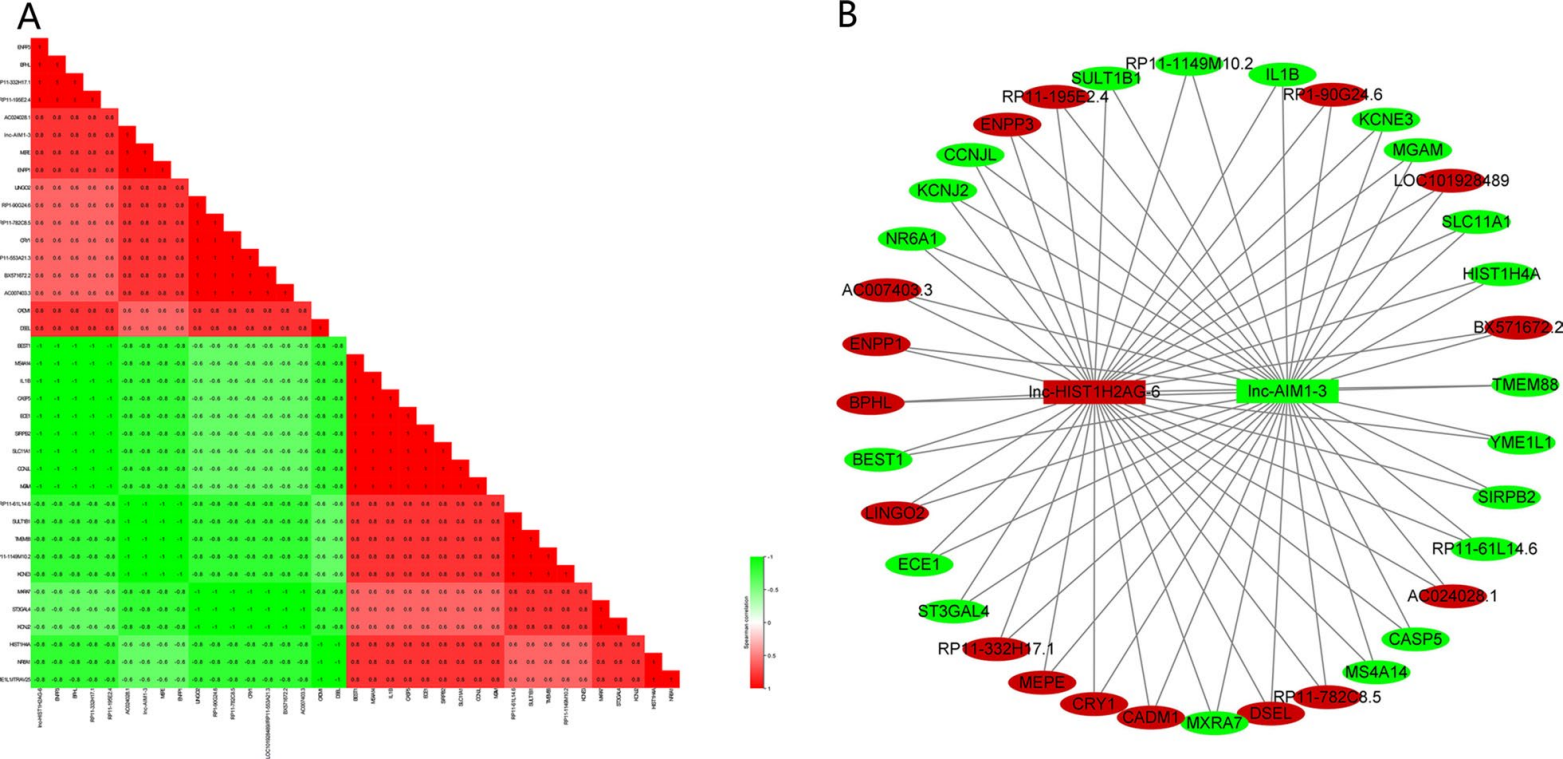
Correlation of lnc-HIST1H2AG-6 and lnc-AIM1-3 with differential expression mRNAs. (**A**) Spearman rank coefficient, red color indicates positive correlation and green color indicates negative correlation (**B**) Interaction network, red color indicates upregulated and green color indicates downregulated in T2DM

**Fig. 3:**
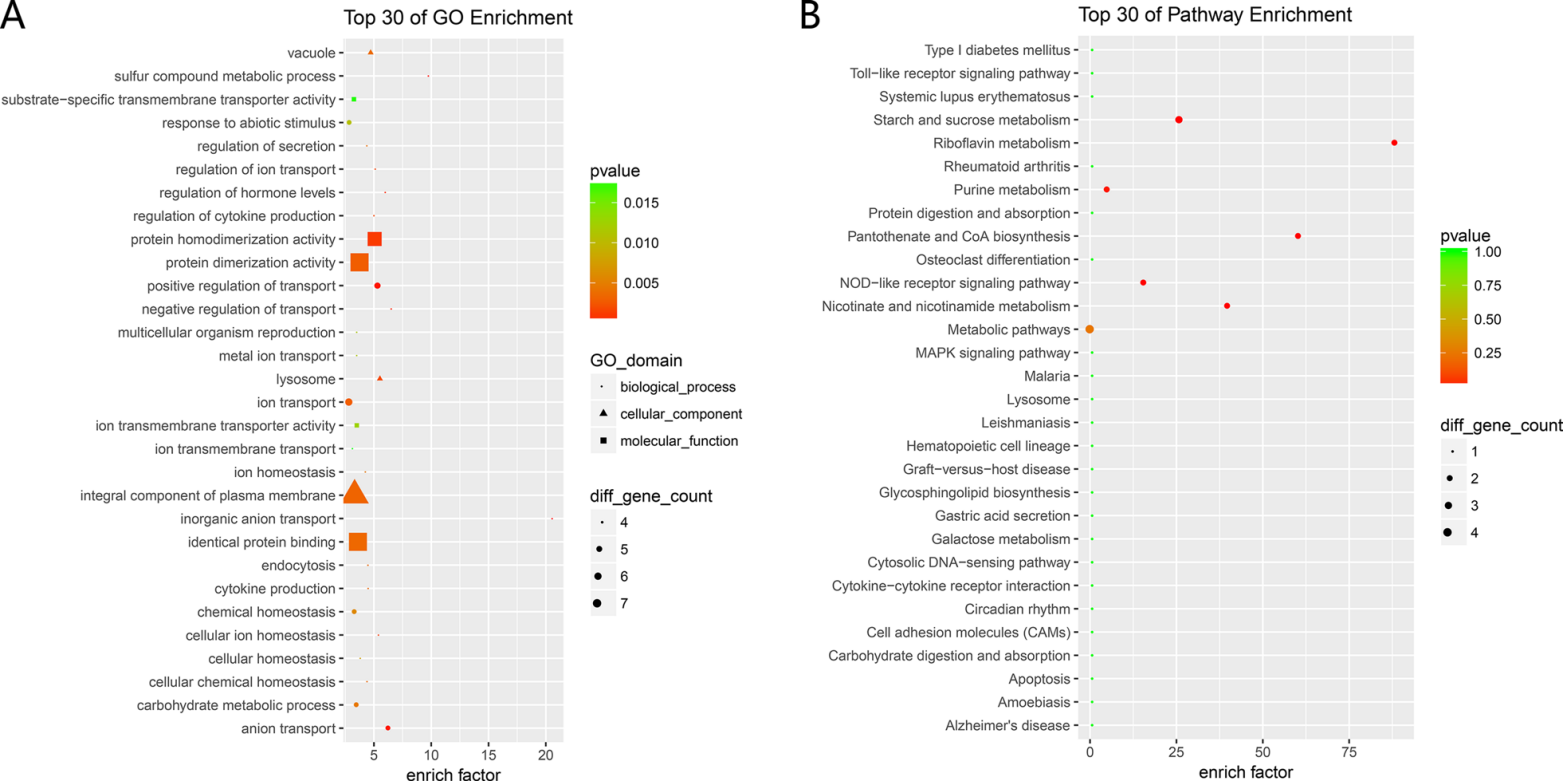
The top 30 enriched GO terms (**A**) and KEGG pathways (**B**) of lnc-HIST1H2AG-6 and lnc-AIM1-3 correlated mRNAs

To better understand the biological functions of the two candidate lncRNAs (lnc-HIST1H2AG-6 and lnc-AIM1-3) and their potential role in T2DM, Spearman’s rank correlation and functional enrichment analysis were performed. Spearman’s rank correlation revealed that the expression levels of lnc-HIST1H2AG-6 and lnc-AIM1-3 were correlated with 36 differentially expressed mRNAs (Fig. 2). Furthermore, lnc-HIST1H2AG-6 and lnc-AIM1-3 related mRNAs were enriched in multiple KEGG pathways (eg, starch and sucrose metabolism, NOD-like receptor signaling pathway, metabolic pathways, and MAPK signaling pathway) and GO terms (eg, cellular metabolic process, immune response and signal transduction). The top 30 KEGG pathway and GO terms are presented in Fig. 3, and the detailed information of KEGG pathway and GO terms is listed in Table 3 and Additional file 3, respectively. The enriched pathways (eg, starch and sucrose metabolism and MAPK signaling pathway) and related genes (eg, *IL1B* and *ENPP1*) have been reported association with T2DM [22–24], which indicated that lnc-HIST1H2AG-6 and lnc-AIM1-3 might play a role in T2DM.

**Table 3:** Enriched pathways of lnc-HIST1H2AG-6N and lnc-AIM1-335 related mRNAs

KEGG pathway	Genes count	Enriched genes
Riboflavin metabolism	2	ENPP3 ENPP1
Pantothenate and CoA biosynthesis	2	ENPP3 ENPP1
Nicotinate and nicotinamide metabolism	2	ENPP3 ENPP1
Starch and sucrose metabolism	3	MGAM ENPP3 ENPP1
NOD-like receptor signaling pathway	2	IL1B CASP5
Purine metabolism	2	ENPP3 ENPP1
Metabolic pathways	4	MGAM ST3GAL4 ENPP3 ENPP1
Osteoclast differentiation	1	IL1B
Carbohydrate digestion and absorption	1	MGAM
Lysosome	1	SLC11A1
Apoptosis	1	IL1B
Rheumatoid arthritis	1	IL1B
Cytokine-cytokine receptor interaction	1	IL1B
Alzheimer's disease	1	IL1B
Type I diabetes mellitus	1	IL1B
Circadian rhythm	1	CRY1
Leishmaniasis	1	IL1B
Systemic lupus erythematosus	1	HIST1H4A
Glycosphingolipid biosynthesis	1	ST3GAL4
Graft-versus-host disease	1	IL1B
Toll-like receptor signaling pathway	1	IL1B
Gastric acid secretion	1	KCNJ2
Amoebiasis	1	IL1B
Galactose metabolism	1	MGAM
Hematopoietic cell lineage	1	IL1B
Cell adhesion molecules (CAMs)	1	CADM1
Malaria	1	IL1B
Cytosolic DNA-sensing pathway	1	IL1B
MAPK signaling pathway	1	IL1B
Protein digestion and absorption	1	KCNE3
African trypanosomiasis	1	IL1B
Prion diseases	1	IL1B
Chagas disease (American trypanosomiasis)	1	IL1B

### Correlations between the two lncRNAs expression and parameters in T2DM

Correlation analysis was used to investigate whether lnc-HIST1H2AG-6 and lnc-AIM1-3 were associated with clinical and laboratory features in our cohort of T2DM. According to our study, lnc-HIST1H2AG-6 was positively correlated with LDL-C and UA (*P* = 0.026 and *P* = 0.021 respectively) and negatively correlated with HOMA-B (*P* = 0.039); lnc-AIM1-3 had a positive correlation with FIN, HOMA-B, and HDL-C (*P* = 0.003, *P* < 0.001, and *P* < 0.001, respectively) and a negative correlation with FPG and HbA1c (*P* < 0.001 and *P* < 0.001, respectively) (Table 4).

**Table 4:** Correlations between expressions of the two candidate lncRNAs and metabolic characteristics

Demographic and metabolic characteristics	lnc-HIST1H2AG-6		lnc-AIM1-3	
	r	P	r	P
Age	0.007	0.945	− 0.380	− 0.734
BMI (kg/m^2^)	0.101	0.338	− 0.930	0.456
SBP (mmHg)	0.192	0.056	− 0.181	− 0.181
DBP (mmHg)	0.108	0.285	− 0.121	− 0.121
FPG (mmol/L)	0.197	0.060	− 0.493	− 0.493
HbA1c (%)	0.172	0.101	− 0.477	− 0.477
FIN (uIu/ml)	− 0.184	0.079	0.327	0.327
HOMA-*β*	− 0.216	0.039	0.507	0.507
HOMA-IR	0.043	0.685	− 0.106	− 0.106
TG (mmol/l)	0.197	0.060	0.021	0.021
LDL-C (mmol/l)	0.274	0.026	− 0.078	− 0.078
HDL-C (mmol/l)	0.050	0.639	0.394	0.394
UA (mmol/L)	0.231	0.021	− 0.062	− 0.062

BMI, body mass index; DBP, diastolic blood pressure; SBP, systolic blood pressure; WBC, white blood cell; FPG, fasting plasma glucose; HbA1c, haemoglobin A1c; FIN, fasting insulin; TG, triglyceride; LDL-C, low density lipoprotein cholesterol; HDL-C, high density lipoprotein cholesterol; UA, urine acid

### Multivariate logistic regression

Using multivariate logistic regression (Table 5), we observed five variables (BMI, LDL-C, HDL-C, lnc-HIST1H2AG-6, and lnc-AIM1-3) that were significantly correlated with T2DM. For example, the expression of lnc-AIM1-3 was negatively correlated with T2DM [*β* =  − 2.54, odds ratio (OR) = 0.071, 95% confidence interval (CI) = 0.029–0.178, *P* = 0.000], and lnc-HIST1H2AG-6 expression was positively associated with T2DM (*β* = 1.756, OR = 5.791, 95% CI = 2.275‒14.739, *P* = 0.000). The results implicated a potential role of lnc-HIST1H2AG-6 and lnc-AIM1-3 in T2DM.

**Table 5:** Multivariate logistic regression analysis to reveal the association of T2DM with different variables including age, BMI, SBP, LDL-C, HDL-C, lnc-HIST1H2AG-6 and lnc-AIM1-3

Factors	β	SE	Wald	P	OR	95% CI
Age (years)	− 0.003	0.020	0.026	0.871	0.997	0.958–1.037
BMI (kg/m^2^)	0.200	0.071	8.006	0.005	1.222	1.063–1.403
SBP (mmHg)	0.026	0.019	7.081	0.176	1.026	0.988–1.066
LDL (mmol/l)	1.014	0.373	7.403	0.007	2.755	1.328–5.718
HDL (mmol/l)	− 3.057	0.941	13.878	0.000	0.030	0.005–0.190
lncRNAHIST1H2AG-6	1.756	0.477	13.575	0.000	5.791	2.275–14.739
lncRNAAIM1-3	− 2.54	0.466	32.18	0.000	0.071	0.029–0.178

BMI, body mass index; SBP, systolic blood pressure; LDL-C, low density lipoprotein cholesterol; HDL-C, high density lipoprotein cholesterol

### Diagnostic potential of lnc-HIST1H2AG-6 and lnc-AIM1-3

**Fig. 4:**
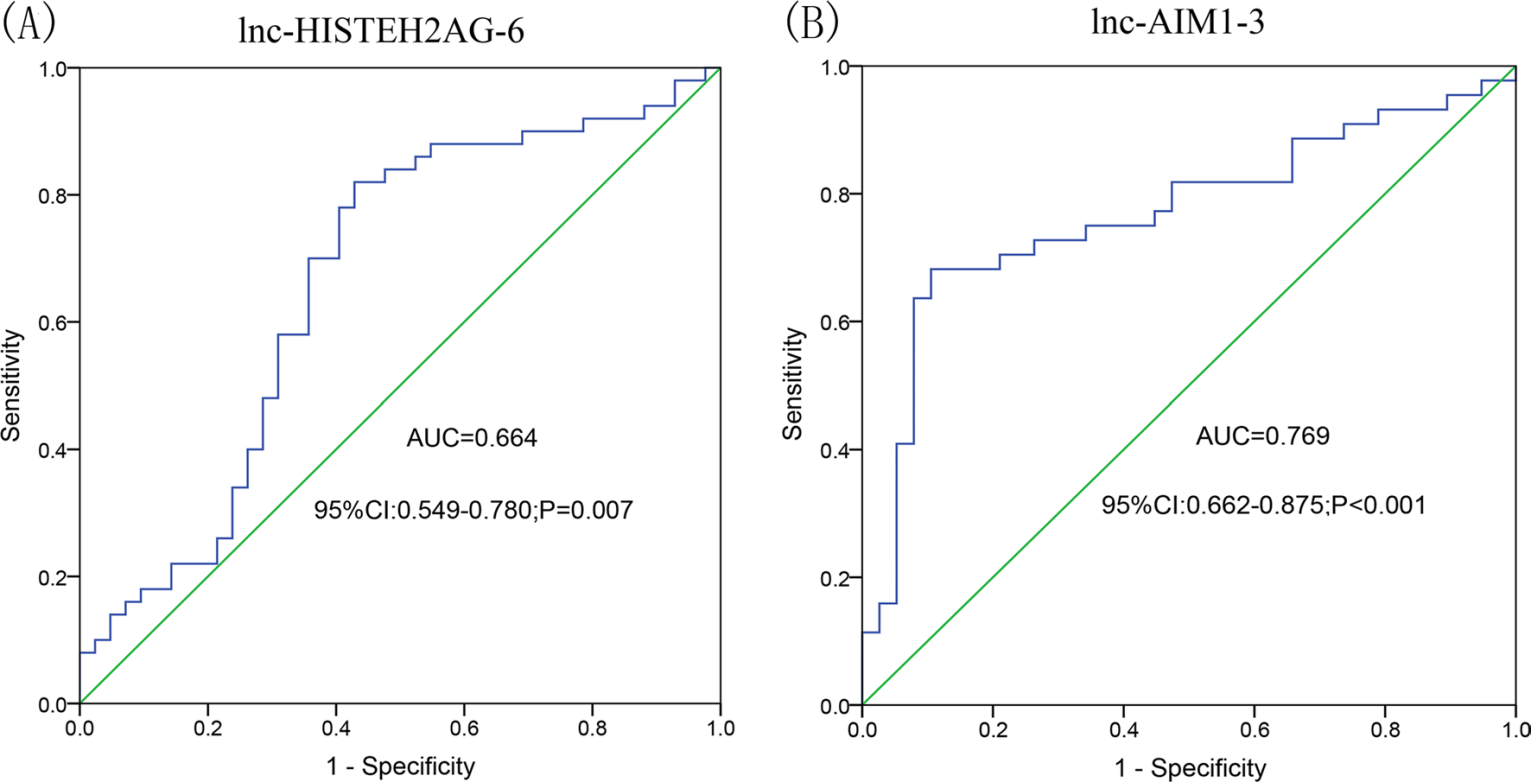
ROC curves of lnc-HIST1H2AG-6 (**A**) and lnc-AIM1-3 (**B**)

To evaluate whether the two lncRNAs could be used to distinguish patients with T2DM and healthy controls, ROC curve analysis was performed and AUC was calculated for lnc-HIST1H2AG-6 and lnc-AIM1-3. As shown in Fig. 4, the AUC for lnc-HIST1H2AG-6 was 0.664 (95% CI = 0.549‒0.780, *P* = 0.007) and that for lnc-AIM1-3 was 0.769 (95% CI = 0.662‒0.875, *P* < 0.001). The results indicated that both lnc-HIST1H2AG-6 and lnc-AIM1-3 might be used to distinguish patients with T2DM and healthy controls.

## Discussion

T2DM is characterized by impaired insulin secretion and IR. The prevalence of T2DM and prediabetes has been increasing rapidly during the previous three decades, especially in developing countries, making it one of the most vital public health challenges. At present, the islet tissue samples of "gold samples" are extremely difficult to obtain clinically; therefore, if the circulating lncRNA can be used as a clinical marker, it will be more valuable for human physiological and pathological conditions, including T2DM. Recently, several lncRNAs have been proved to be widely involved in many biological processes, such as cell proliferation, differentiation, cycle regulation, and apoptosis [25–27]. It was reported that circulating lncRNAs are potential diagnostic and prognostic factors in diabetes [28, 29]. The lncRNAs KCNQ1OT1 and ANRIL have been identified as genetic susceptibility loci associated with T2DM [30, 31]. Imamura, et al. revealed that rs7656416 near CTBP1-AS2 were significantly associated with T2DM [32]. Willer et al. showed that rs10904908 at the VIM-CUBN locus on chromosome 10 was associated with lipid levels [33], indicating that lncRNAs might play an important role in regulating gene expression and could be explored as a specific biomarker for T2DM diagnosis and prediction.

PBMCs are a component of the blood that mainly consists of monocytes or macrophages and lymphocytes. In the human genome, PBMCs show a large proportion (about 80%) of the coding genes and are easy to determine as a marker in human diseases [34, 35]. Recent research on PBMC has indicated that the deregulation of some lncRNAs might be associated with T2DM. Mohamadi et al. have revealed that the expression of SNHG17 and TTC28AS1 in PBMCs was downregulated in patients with T2DM compared with that in healthy controls, indicating that the downregulation of the lncRNAs TTC28-AS1 and SNHG17 in patients with T2DM was associated with T2DM susceptibility [36]. Similarly, Sathishkumar et al. provided the first preliminary evidence that the expression of the lncRNAs THRIL and SALRNA1 in PBMC was decreased in patients with T2DM and negatively correlated with hyperglycemia, senescence, and inflammation. Omidvar et al. reported that decreased levels of VIM-AS1 and CTBP1-AS2 in PBMCs were associated with diabetes in an Iranian population, implying that the expression levels of lncRNA VIM-AS1 and lncRNA CTBP1-AS2 might be associated with T2DM susceptibility [37]. Thus, we reasoned that DEGs in the PMBCs of T2DM and healthy controls might offer a better understanding of the underlying pathology of T2DM and might provide novel therapeutic targets and biomarkers.

In the present study, microarray analysis was conducted to screen the lncRNA and mRNA expression profiles in PBMCs of newly diagnosed patients with T2DM and healthy controls; 55 lncRNAs (18 upregulated and 37 downregulated) and 36 mRNAs (16 upregulated and 20 downregulated) were differentially expressed in patients with T2DM compared with healthy controls. The expression levels of two candidate lncRNAs (lnc-HIST1H2AG-6 and lnc-AIM1-3) were validated by a large-scale qRT-PCR experiment. To better understand the biological function of lnc-HIST1H2AG-6 and lnc-AIM1-3, their correlated mRNAs were screened and functional enrichment analysis was performed. Our results indicated that lnc-HIST1H2AG-6 and lnc-AIM1-3 were correlated with 36 differentially expressed mRNAs (such as *CADM1, IL1B,* and *ENPP1*), which might be involved in many KEGG pathways (such as starch and sucrose metabolism and MAPK signaling pathway). It has been reported that with CADM1, an immunoglobulin superfamily member, ectodomain shedding contributed to blood glucose dysregulation in T2DM [38]. Gene variants or polymorphisms of *IL1B* and *ENP1* have been found to correlate with T2DM in many human studies [39–41]. Moreover, starch and sucrose metabolism and MAPK signaling pathways have been implicated in the development of T2DM in multiple animal models and human studies [42, 43]. The findings of the present study suggested that lnc-HIST1H2AG-6 and lnc-AIM1-3 play an important role in T2DM; thus, further investigation should be performed.

Correlations between the two candidate lncRNAs (lnc-HIST1H2AG-6 and lnc-AIM1-3) and different variables, including FPG, HbA1c, FIN and HOMA-B were identified in our cohort. Our results indicated that lnc-HIST1H2AG-6 was positively correlated with FPG, triglyceride, LDL-C, and UA and negatively correlated with HOMA-B. However, lnc-AIM1-3 exhibited a positive correlation with FIN, HOMA-B, and HDL-C and a negative correlation with SBP, FPG, and HbA1c. These results were similar to a previous study which reported that the lncRNAs (n342533, n335556, and n336109) were closely correlated to BMI, FPG, 2hPG, HbA1c, HOMA-IR, alanine transaminase, HDL-C, and HOMA-B of patients with T2DM and healthy controls [44]. On the basis of these correlations between the two candidate lncRNAs and metabolic characteristics (FPG, HbA1c, LDL-C, HDL-C, FINs, and HOMA-B), we could conclude that lnc-HIST1H2AG-6 and lnc-AIM1-3 might be involved in glucose regulation, lipid homeostasis, and the secretion of insulin. Impaired function of islet *β* cells and reduced insulin sensitivity are the two remarkable characteristics of T2DM. Accumulating evidence suggests that lncRNAs could affect apoptosis and insulin secretion in the pancreatic *β* cells [15, 17, 45]. Recently, increasing evidence has indicated that lncRNAs may act as a link between insulin signaling and IR [46]. Ruan et al. found that the overexpression of lncRNA-p3134 could reduce the apoptosis of islet cells and increase the level of glucose-stimulated insulin by positively regulating the PI3K/AKT/mTOR (phosphoinositide-3-kinase/protein kinase B/mammalian target of rapamycin) signaling pathway [47]. As mentioned above, lnc-HIST1H2AG-6 and lnc-AIM1-3 might be involved in multiple KEGG pathways; further functional studies should be performed to identify their underlying mechanisms in T2DM.

Furthermore, our multivariate logistic regression analysis revealed that some variables (such as BMI, LDL-C, and HDL-C) were significantly correlated with T2DM, which was consistent with previous studies to some extent. For example, Mohamadi, et al. showed that the increase in FBG, SBP, and BMI was associated with an increased likelihood of exhibiting T2DM [36]. Pan et al. have reported that elevated LDL-C levels significantly increased the risk of T2DM [48]. Some reports have shown that hypertension was an independent risk factor for T2DM [49, 50]. However, we observed that the association of SBP with T2DM was not statistically significant. We believed that this could be due to the small sample size of our study or the source of our samples, as lncRNAs displayed tissue-specific patterns of expression [51]. It is worth noting that our results suggested that the expression levels of lnc-HIST1H2AG-6 and lnc-AIM1-3 are significantly correlated with T2DM, which might be used to distinguish patients with T2DM and healthy controls. Further investigation may obtain novel lncRNA biomarkers of T2DM in the future.

Our study has the following limitations: (1) The alterations observed in the PBMCs might be just appearances of the tissue-specific and heterogeneous actions of lncRNAs. (2) This was a single-center study with a relatively small sample size, which needed further large-scale validation in multicenters with comprehensive investigation in the future. (3) With respect to the microarray analysis, there were only two samples in each group, and only two lncRNAs have been verified; the relationship between the other 53 differentially expressed lncRNAs and diabetes warrants further exploration. (4) Based on the present results, it is not possible to infer the causal relationship between these two lncRNAs changes and type 2 diabetes; repeated and prospective follow-up studies are needed. Next, we will further study the role of both lncRNAs in the pathogenesis of diabetes in insulinoma cells and diabetic mice.

## Conclusion

The profiles of lncRNA and mRNA were significantly changed in the patients with T2DM compared with healthy controls. The expression levels of lnc-HIST1H2AG-6 and lnc-AIM1-3 were significantly correlated with some features of T2DM, which might be used to distinguish patients with T2DM and healthy controls and serve as potential novel biomarkers for diagnosis in the future.

## Supplementary Information

**Additional file 1.** Differentially expressed lncRNAs in patients with T2DM compared with healthy controls.

**Additional file 2.** Differentially expressed mRNAs in patients with T2DM compared with healthy controls.

**Additional file 3.** Detailed information of enriched GO terms of lnc-HIST1H2AG-6 and lnc-AIM1-3 correlated mRNAs.

## Data Availability

The datasets generated and/or analysed during the current study are available in the GEO repository, https://www.ncbi.nlm.nih.gov/geo/query/acc.cgi?acc=GSE168437. (Accession number: ezmfqucejvsjfwx).

## Abbreviations

T2DM: Type 2 diabetes mellitus
lncRNAs: Long noncoding RNA
qRT-PCR: Quantitative real-time polymerase chain reaction
KEGG: Kyoto Encyclopedia of Genes and Genomes
GO: Gene ontology
ROC: Receiver operating characteristic
DM: Diabetes mellitus
IDF: International Diabetes Federation
ncRNAs: Noncoding RNAs
siRNAs: Small interfering RNAs
circRNA: Circular RNA
piRNA: Piwi-interacting RNA
MEG3: Maternally expressed gene 3
TUG1: Taurine raised gene 1
IR: Insulin resistance
Blnc1: Brown fat lncRNA1
WHO: World Health Organization
BMI: Body mass index
TC: Total cholesterol
TG: Triglyceride
HDL-C: High-density lipoprotein cholesterol
LDL-C: Low-density lipoprotein cholesterol
UA: Uric acid
HbA1C: Hemoglobin A1c
HOMA-IR: Homeostasis model assessment-IR
PBMCs: Peripheral blood mononuclear cells
DEGs: Differentially expressed genes
SD: Standard deviation
