# Supplementary material for: Deregulation of lncRNA HIST1H2AG-6 and AIM1-3 in peripheral blood mononuclear cells is associated with newly diagnosed type 2 diabetes
Source: BMC Med Genomics. 2021 Jun 6;14:149. doi: 10.1186/s12920-021-00994-z (PMC8182924; doi:10.1186/s12920-021-00994-z)
Supplement: Supplementary file 1 — Additional file 1. Differentially expressed lncRNAs in patients with T2DM compared with healthy controls. [file 12920_2021_994_MOESM1_ESM.docx]

Additional file 1 Differently expressed lncRNAs in the T2DM group as compared to that in the control group.

| Probe Set ID | p | FC (abs) | Regulation | GeneSymbol | Chr | strand |
| --- | --- | --- | --- | --- | --- | --- |
| TC1100001940.oe.1 | 0.049639735 | 4.5387616 | down | lnc-SOX6-6 | chr11 | - |
| TC0200000454.oe.1 | 0.003045324 | 4.1086197 | down | lnc-TMEM178-6 | chr2 | + |
| TC0700001088.oe.1 | 0.048202753 | 4.0329914 | down | lnc-CDHR3-6 | chr7 | + |
| TC0200003843.oe.1 | 0.01780926 | 3.3764462 | down | lnc-IL1A-3 | chr2 | - |
| TC0700001412.oe.1 | 0.037610922 | 3.335991 | down | lnc-RP11-1220K2.2.1-7 | chr7 | + |
| TC0300003361.oe.1 | 0.014910806 | 2.8787036 | down | lnc-BCL6-1 | chr3 | - |
| TC0200001545.oe.1 | 0.049992505 | 2.8554623 | down | lnc-ARHGAP15-8 | chr2 | + |
| TC0100004530.oe.1 | 0.002429841 | 2.5750308 | down | OTTHUMG00000041315 | chr1 | - |
| TC1900002690.oe.1 | 0.036802515 | 2.5488126 | down | lnc-LILRA5-2 | chr19 | - |
| TC1700000176.oe.1 | 0.04120358 | 2.482007 | down | RP11-1099M24.6 | chr17 | + |
| TC10000326.hg.4 | 0.017677357 | 2.4608986 | down | FAM170B-AS1 | chr10 | + |
| TC0400000866.oe.1 | 0.021917617 | 2.433699 | down | lnc-ALPK1-4 | chr4 | + |
| TC1100002858.oe.1 | 0.009113951 | 2.4151278 | down | lnc-FAM76B-1 | chr11 | - |
| TC0200002688.oe.1 | 0.023761585 | 2.3761938 | down | lnc-KIDINS220-11 | chr2 | - |
| TC1900000951.oe.1 | 0.017834375 | 2.3607285 | down | NONHSAG025880 | chr19 | + |
| TC0700001084.oe.1 | 0.001887456 | 2.3500566 | down | RP11-22N19.2 | chr7 | + |
| TC1200001865.oe.1 | 0.00749196 | 2.2908041 | down | lnc-KLRB1-3 | chr12 | - |
| TC0500003166.oe.1 | 0.0229978 | 2.2533486 | down | lnc-CCNJL-2 | chr5 | - |
| TC1000001989.oe.1 | 0.038672354 | 2.2470164 | down | lnc-EGR2-5 | chr10 | - |
| TC1000001429.oe.1 | 0.02355063 | 2.2236817 | down | lnc-CYP2E1-10 | chr10 | + |
| TC0600001210.oe.1 | 0.022809027 | 2.151183 | down | lnc-AIM1-3 | chr6 | + |
| TC0X00000106.oe.1 | 0.04123587 | 2.2127197 | down | lnc-NHS-3 | chrX | + |
| TC1200000423.oe.1 | 0.002540745 | 2.2006097 | down | lnc-TSPAN11-7 | chr12 | + |
| TC1700002910.oe.1 | 0.049848627 | 2.194781 | down | RP11-649A18.4 | chr17 | - |
| TC0600000405.oe.1 | 0.014088072 | 2.168295 | down | lnc-GMNN-4 | chr6 | + |
| TC0300002219.oe.1 | 0.013706771 | 2.1630838 | down | lnc-ZNF445-4 | chr3 | - |
| TC0200002227.oe.1 | 0.005449599 | 2.1532445 | down | lnc-C2orf62-1 | chr2 | + |
| TC1700000095.oe.1 | 0.03171964 | 2.1495085 | down | lnc-ARRB2-3 | chr17 | + |
| TC1100003063.oe.1 | 6.99E-04 | 2.1162577 | down | lnc-MPZL3-1 | chr11 | - |
| TC1700002133.oe.1 | 0.012720627 | 2.1071367 | down | RP11-68I3.10 | chr17 | - |
| TC0100004430.oe.1 | 0.007865801 | 2.0765007 | down | RP11-196G18.22 | chr1 | - |
| TC0200002229.oe.1 | 0.021880493 | 2.0477283 | down | lnc-CTDSP1-1 | chr2 | + |
| TC0200002222.oe.1 | 2.70E-04 | 2.0449667 | down | lnc-ARPC2-1 | chr2 | + |
| TC0200003862.oe.1 | 0.011057267 | 2.0427663 | down | lnc-SLC35F5-11 | chr2 | - |
| TC1900001449.oe.1 | 0.032805216 | 2.0380943 | down | lnc-TRIM28-12 | chr19 | + |
| TC0500001965.oe.1 | 0.006184289 | 2.0258436 | down | lnc-MYO10-6 | chr5 | - |
| TC0300000936.oe.1 | 0.0483222 | 2.0142136 | down | lnc-SLC35A5-1 | chr3 | + |
| TSUnmapped00002035.oe.1 | 0.017019017 | 2.0014071 | down | lnc-MBOAT7-3 | --- | --- |
| TC0600000460.oe.1 | 0.005657883 | 4.754367 | up | lnc-HIST1H2AG-6 | chr6 | + |
| TC1000001451.oe.1 | 0.04136468 | 7.385878 | up | lnc-IDI1-5 | chr10 | - |
| TC2100000551.oe.1 | 0.022648485 | 3.9375045 | up | lnc-LIPI-4 | chr21 | - |
| TSUnmapped00001104.oe.1 | 0.04305197 | 3.4949276 | up | lnc-PPIAL4G-5 | --- | --- |
| TC1800000207.oe.1 | 0.010743975 | 3.3245566 | up | lnc-MC5R-11 | chr18 | + |
| TC0400001874.oe.1 | 0.010726228 | 3.1234994 | up | lnc-OCIAD2-11 | chr4 | - |
| TC0500000612.oe.1 | 0.049826253 | 3.0443666 | up | lnc-GTF2H2C-3 | chr5 | + |
| TC0500002003.oe.1 | 0.036466528 | 3.0128927 | up | lnc-CDH12-2 | chr5 | - |
| TC2100000019.oe.1 | 0.042963587 | 2.6996317 | up | lnc-CR381653.1-5 | chr21 | + |
| TC0600002908.oe.1 | 0.047074277 | 2.267439 | up | lnc-TRAF3IP2-7 | chr6 | - |
| TC0600001426.oe.1 | 0.0202517 | 2.2427917 | up | lnc-ARG1-3 | chr6 | + |
| TC21000281.hg.4 | 0.014507043 | 2.1673362 | up | ANKRD20A11P | chr21 | - |
| TC0400001872.oe.1 | 0.017255561 | 2.158867 | up | lnc-OCIAD2-9 | chr4 | - |
| TC0100002267.oe.1 | 0.045740847 | 2.125265 | up | lnc-CDC73-10 | chr1 | + |
| TSUnmapped00000285.oe.1 | 0.01942654 | 2.114321 | up | lnc-SYNCRIP-3 | --- | --- |
| TC0500000492.oe.1 | 0.022146102 | 2.0785868 | up | lnc-C5orf35-3 | chr5 | + |
| TC21000277.hg.4 | 0.039914582 | 2.0230405 | up | C21orf15 | chr21 | - |
