# Supplementary material for: Deregulation of lncRNA HIST1H2AG-6 and AIM1-3 in peripheral blood mononuclear cells is associated with newly diagnosed type 2 diabetes
Source: BMC Med Genomics. 2021 Jun 6;14:149. doi: 10.1186/s12920-021-00994-z (PMC8182924; doi:10.1186/s12920-021-00994-z)
Supplement: Supplementary file 2 — Additional file 2. Differentially expressed mRNAs in patients with T2DM compared with healthy controls. [file 12920_2021_994_MOESM2_ESM.docx]

Additional file 2 Differently expressed mRNAs in the T2DM group as compared to that in the control group.

| Probe Set ID | p | FC (abs) | Regulation | GeneSymbol | Chr | strand |
| --- | --- | --- | --- | --- | --- | --- |
| TC20001743.hg.4 | 0.033894945 | 3.5294414 | down | SIRPB2 | chr20 | - |
| TC06001348.hg.4 | 0.0245728 | 3.382234 | down | HIST1H4A | chr6 | - |
| TC05002007.hg.4 | 0.005262666 | 3.3819904 | down | CCNJL | chr5 | - |
| TC17000813.hg.4 | 0.0219209 | 3.1537685 | down | KCNJ2 | chr17 | + |
| TC11002247.hg.4 | 0.04470819 | 3.0258062 | down | CASP5 | chr11 | - |
| TC02002219.hg.4 | 0.016089123 | 2.989234 | down | IL1B | chr2 | - |
| TC07000899.hg.4 | 0.026443802 | 2.9492216 | down | MGAM | chr7 | + |
| TC01003294.hg.4 | 0.020427436 | 2.787971 | down | RP11-61L14.6 | chr1 | - |
| TC17000118.hg.4 | 0.026505573 | 2.7874625 | down | TMEM88 | chr17 | + |
| TC08000517.hg.4 | 0.0174269 | 2.7258916 | down | RP11-1149M10.2 | chr8 | + |
| TC11000544.hg.4 | 0.032174777 | 2.694742 | down | BEST1 | chr11 | + |
| TC14000108.hg.4 | 0.023582498 | 2.6892505 | down | YME1L1//TRAV25 | chr14 | + |
| TC02001300.hg.4 | 0.012062945 | 2.3747725 | down | SLC11A1 | chr2 | + |
| TC17001905.hg.4 | 0.011421889 | 2.3347754 | down | MXRA7 | chr17 | - |
| TC11002082.hg.4 | 0.00695067 | 2.2844892 | down | KCNE3 | chr11 | - |
| TC09001580.hg.4 | 0.011654287 | 2.219574 | down | NR6A1 | chr9 | - |
| TC04001267.hg.4 | 0.013112129 | 2.200323 | down | SULT1B1 | chr4 | - |
| TC01002322.hg.4 | 0.011536205 | 2.0880852 | down | ECE1 | chr1 | - |
| TC11001174.hg.4 | 0.03948202 | 2.0592651 | down | ST3GAL4 | chr11 | + |
| TC11003452.hg.4 | 0.021283757 | 2.0523777 | down | MS4A14 | chr11 | + |
| TC01003068.hg.4 | 0.018785646 | 4.6091514 | up | BX571672.2 | chr1 | - |
| TC05001456.hg.4 | 0.02185639 | 3.845806 | up | RP11-195E2.4 | chr5 | - |
| TC11002316.hg.4 | 0.012733777 | 3.3049326 | up | CADM1 | chr11 | - |
| TC01003070.hg.4 | 0.046179034 | 2.913829 | up | RP11-782C8.5 | --- | --- |
| TC06000984.hg.4 | 0.04533208 | 2.7412539 | up | ENPP3 | chr6 | + |
| TC09000993.hg.4 | 0.006137595 | 2.6695902 | up | LINGO2 | chr9 | - |
| TC06001877.hg.4 | 0.00173477 | 2.5303206 | up | LOC101928489 | chr6 | - |
| TC06000985.hg.4 | 0.00715941 | 2.5227015 | up | ENPP1 | chr6 | + |
| TC04000476.hg.4 | 0.00757026 | 2.4515812 | up | MEPE | chr4 | + |
| TC22000242.hg.4 | 0.019078169 | 2.2938757 | up | RP1-90G24.6 | chr22 | + |
| TC06000024.hg.4 | 0.044166658 | 2.2412503 | up | BPHL | chr6 | + |
| TC01001478.hg.4 | 0.0485422 | 2.1943552 | up | RP11-332H17.1 | chr1 | + |
| TC02000389.hg.4 | 0.022603028 | 2.180685 | up | AC007403.3 | chr2 | + |
| TC12001925.hg.4 | 0.044988487 | 2.1428928 | up | CRY1 | chr12 | - |
| TC18000564.hg.4 | 0.018865405 | 2.0649137 | up | DSEL | chr18 | - |
| TC07001103.hg.4 | 0.00977979 | 2.0317235 | up | AC024028.1 | chr7 | - |
